# Co-occurrence of obesogenic behaviors and their implications for mental health during the COVID-19 pandemic: a study with university students

**DOI:** 10.1186/s12889-024-19031-6

**Published:** 2024-06-14

**Authors:** Bruna Carolina Rafael Barbosa, Raquel de Deus Mendonça, Elaine Leandro Machado, Adriana Lúcia Meireles

**Affiliations:** 1 Postgraduate Program in Health and Nutrition, School of Nutrition, Federal University of Ouro Preto, Ouro Preto, Brazil; 2 Department of Clinical and Social Nutrition, School of Nutrition, Federal University of Ouro Preto, Ouro Preto, Brazil; 3 Department of Preventive and Social Medicine, Federal University of Minas Gerais, Belo Horizonte, Brazil; 4 Research and Study Group on Nutrition and Public Health, Federal University of Ouro Preto, Ouro Preto, Brazil

**Keywords:** COVID-19, Anxiety, Depression, Health risk behaviors, Fruit and vegetable consumption, Ultra-processed food, Sedentary behavior, Physical inactivity, Students

## Abstract

**Background:**

The university years are a critical period for young adults, as they are more exposed to obesogenic behaviors and experience stressful situations that compromise their mental health. This study aims to estimate the prevalence of anxiety and depression symptoms and evaluate the association between the combined occurrence of obesogenic behaviors among university students.

**Methods:**

A cross-sectional study was conducted on students from a public university in Brazil during the COVID-19 pandemic. Data were collected from July to August 2020 using an online questionnaire. The outcome variables (anxiety and depression symptoms) were assessed using the Depression, Anxiety and Stress Scale-21 (DASS-21). The co-occurrence of obesogenic behaviors was measured based on irregular consumption of fruits and vegetables, frequent consumption of ultra-processed foods, physical inactivity during leisure time, and sedentary behavior. A Venn diagram was used for the exploratory analysis. To verify the association between the outcome and explanatory variables, a directed acyclic graph model was constructed, and multivariate logistic regression was performed to calculate odds ratios (ORs) and 95% confidence intervals (95%CIs).

**Results:**

A total of 1,353 students aged 18–24 years participated in this study. Symptoms of anxiety and depression were present in 46.1% and 54.6% of the participants, respectively. The most prevalent combination of obesogenic behaviors was frequent consumption of ultra-processed foods, physical inactivity during leisure time, and sedentary behavior (17.2%). The greater the number of simultaneous obesogenic behaviors, the higher the chance to present symptoms of anxiety [OR: 2.81 (95%CI: 1.77–4.46)] and depression [OR: 3.46 (95%CI: 2.20–5.43)].

**Conclusion:**

These findings reinforce the need to take actions to promote mental health in the university environment in conjunction with programs to promote a healthy lifestyle and improve the physical and mental well-being of students.

## Introduction

The COVID-19 pandemic affected individuals’ physical and mental well-being, causing behavioral changes and mental health issues such as anxiety and depression [1]. Although the effects of the pandemic were felt by the general population, emerging evidence demonstrates that university students are among the most affected [2, 3], due to the suspension of in-person classes and the inclusion of remote teaching added to feelings of fear, worry and stress [4, 5].

Even before the pandemic, the mental health of young adults, typically aged between 18 and 25 years, was a public health concern, as this group has higher levels of mental disorders than individuals in other age groups [2, 3, 6, 7], in addition to being exposed to various psychosocial stressors [8]. A pre-pandemic study estimated the prevalence of major depressive episodes at 32.0% among undergraduate students [9], and concerning figures can also be observed during the COVID-19 pandemic. Barbosa and collaborators [10] using the Depression, Anxiety, and Stress Scale (DASS-21) in a study conducted with students from eight universities in Brazil, observed that 59.7% (95%CI: 58.7–60.7) of participants presented symptoms of anxiety, and 63.0% (95%CI: 62.0–64.0) presented symptoms of depression.

Young adulthood is also considered a potentially important phase in which patterns of behavior that are harmful to health are shaped and developed [11–13]. Admission to university usually occurs at this age, a period that results in several changes in the life of the individual and the environment in which they live, whether in the leisure, domestic, or work environment [14]. Thus, the university context plays a fundamental role in the health-disease process, since university students are more likely to adopt obesogenic behaviors that can influence health in the medium and long terms, in addition to contributing to the development of obesity [1, 14, 15].

In recent decades, the literature has highlighted a paradigm shift in understanding the causes of obesity, focusing on behaviors deemed obesogenic [16]. Obesogenic behaviours are defined as those promoting or contributing to obesity through unhealthy diets through, including the high consumption of ultra-processed foods, low levels of physical activity, high levels of sedentary behaviour (for example high screen time) [17, 18]. The primary cause of obesity is an energy imbalance between calories consumed and calories expended by the individual, driven by obesogenic behaviors. These, in turn, are influenced by a range of individual, biological, genetic, and psychological factors, such as knowledge, motivation, and capability [16, 19].

Despite being a topic that has been widely explored in research on adult populations [20], few studies in Brazil have investigated the clustering of multiple obesogenic behaviors among young adults, mainly in populations of university students [21–23]. Additionally, few studies have assessed how the co-occurrence of obesogenic behaviors is related to symptoms of anxiety and depression among university students, especially during the COVID-19 pandemic. Studies suggest that individuals with mental disorders have a higher prevalence of obesogenic behaviors than the general population [11, 15].

In view of this, this study contributes to understanding the association between multiple obesogenic behaviors and their effects on mental health during the pandemic by using a methodology that considers these behaviors in a grouped manner. Thus, the objective of this study was to estimate the prevalence of anxiety and depression symptoms and assess the association between the co-occurrence of obesogenic behaviors and mental disorders among university students during the initial months of the COVID-19 pandemic.

## Methods

### Study design

This is a cross-sectional study under the project “Effect of the COVID-19 pandemic on mental and nutritional health and on the home food environment of the academic community: longitudinal evaluation (PADu-COVID)” carried out on undergraduate students and employees from a Brazilian university during the COVID-19 pandemic.

### Study population and sample

All students enrolled in on-site and distance undergraduate courses in life sciences, exact sciences, and human, social, and applied sciences were invited to participate in the survey, being considered eligible 11.743 students.

Students who met the following inclusion criteria participated in the study: aged 18 years or older, regularly enrolled in the undergraduate courses evaluated in the study, and completed the questionnaire within four weeks of sending the invitation.

**Fig. 1:**
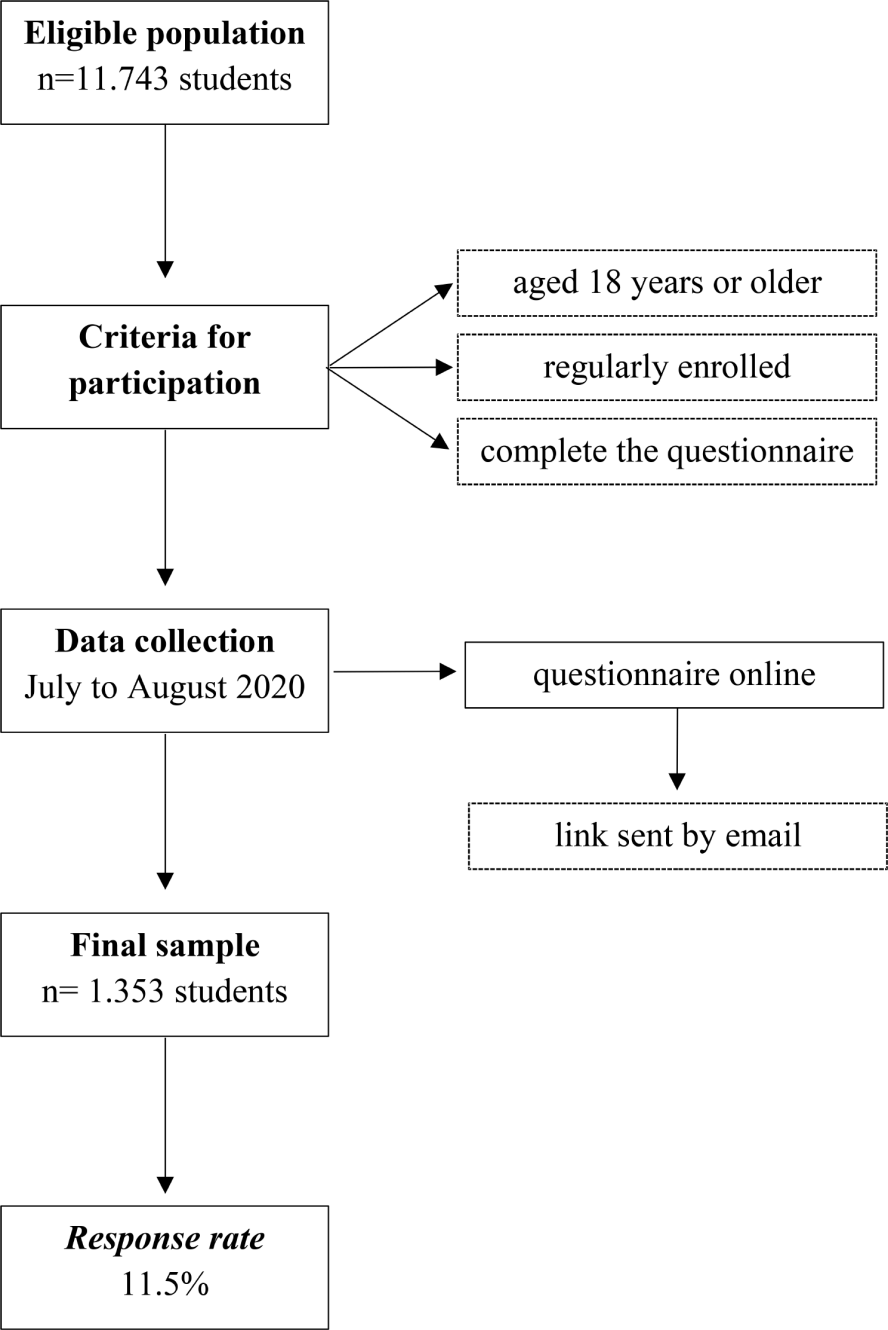
Flowchart of the study population and sample

The PADu-COVID baseline dataset was composed of 1.353 students in remote teaching due to the covid-19 pandemic, with a response rate of 11.5%, based on eligible participants for the study. Figure 1 presents the study methods.

### Data collection

Data were collected from July to August 2020 using a self-administered questionnaire available on Google Forms. The participants received an invitation text and a link to the questionnaire via e-mail. Participation in the study started when the students accessed the questionnaire, subject to agreement with the Free and Informed Consent Form, which was presented electronically and was available for download.

Four reminders were sent, once per week and on alternate days, to students who did not respond to the questionnaire. Refusal to participate in the study was indicated by non-response after two weeks of sending the last reminder. The questionnaire assessed sociodemographic characteristics, lifestyle habits, and health conditions.

### Study variables

#### Outcome variables: symptoms of anxiety and depression

Symptoms of anxiety and depression were assessed using the DASS-21, which was adapted and validated for the Portuguese language by Vignola and Tucci. The DASS-21 consists of three subscales, comprising seven items each, which assess the symptoms of anxiety, depression, and stress presented by individuals in the last week [24]. The subscale items are divided as follows: questions 3, 5, 10, 13, 16, 17, and 21 refer to the depression scale; 2, 4, 7, 9, 15, 19, and 20 refer to the anxiety scale; and 1, 6, 8, 11, 12, 14, and 18 refer to the stress scale.

Responses to the items were scored on a four-point Likert scale ranging from 0 (not applicable) to 3 (applied a lot or most of the time). For the final score, the item values for each subscale are summed and multiplied by two to match the original scale score (DASS-42) [24, 25]. Based on this final score, cutoff points are assumed that allow the classification of depression, anxiety, and stress symptoms into the categories “normal,” “mild,” “moderate,” “severe,” and “extremely severe” (Chart 1).

Chart 1 Instructions for summing the individual scores for each response on the DASS-21 scale.

**Table:** 

Symptom classification	Depression	Anxiety	Stress
Normal	0–9	0–7	0–14
Mild	10–13	8–9	15–18
Moderate	14–20	10–14	19–25
Severe	21–27	15–19	26–33
Extremely severe	≥ 28	≥ 20	≥ 34

Source Adapted from VIGNOLA, Rose Claudia Batistelli; TUCCI, Adriana Marcassa. Adaptation and validation of the depression, anxiety and stress scale (DASS) to Brazilian Portuguese. Journal of affective disorders, v.155, p.104–109, 2014

In this study, only anxiety and depression symptoms were evaluated. The variables were reclassified, with the categories “moderate,” “severe,” and “extremely severe” indicating the presence of symptoms and “normal” and “mild” indicating the absence of symptoms.

### Explanatory variables: co-occurrence of obesogenic behaviors

The co-occurrence of obesogenic behaviors was evaluated based on the sum of four behaviors: irregular consumption of fruits and vegetables, frequent consumption of ultra-processed foods, physical inactivity during leisure time, and sedentary behavior. The responses obtained resulted in no behavior for the four obesogenic behaviors.

The consumption of fruits and vegetables (FV) was estimated based on questions adapted from The Surveillance System of Risk and Protective Factors for Chronic Diseases by Telephone Survey (Vigitel), a population-based cross-sectional survey conducted by the Brazilian Ministry of Health aiming to continuously monitor the prevalence and distribution of noncommunicable diseases and their major associated risk and protective factors among adults (aged 18 years or older) in the 26 Brazilian state capitals and the Federal District [26, 27].

The questions were: “In the last 30 days, how many days a week did you usually eat vegetables (lettuce, tomato, cabbage, carrots, chayote, eggplant, zucchini – not consider potatoes, cassava or yams)?” and “In the last 30 days, how many days a week did you usually eat fruit?”. The questions in this manuscript were adapted from Vigitel questions regarding the frequency of food consumption. Vigitel only assesses “On how many days of the week” the participant consumes fruits and vegetables, for example. In this study, we evaluated food consumption for the “last 30 days”, thus allowing the analysis of the last month.

FV consumption was investigated in terms of frequency: never or almost never; 1–2 days a week; 3–4 days a week; 5–6 days a week; and every day, including Saturdays and Sundays. FV consumption was analyzed as a continuous variable using the mean frequency, and responses were coded as 0 days, 1.5 days, 3.5 days, 5.5 days, and 7 days. FV consumption ≥ 5 days a week was considered regular consumption, and FV consumption ≤ 4 days a week was considered irregular consumption [26, 28].

The frequency of consumption of ultra-processed foods was measured using the question, “In the last 30 days, how many days a week did you usually eat/drink…?”, and the answer options were the same as the questions about FV consumption. The ultra-processed foods investigated were “packaged” snacks, such as chips, sweet biscuits (filled cookies), sweets (chocolate, ice cream, and gelatin), and sugary drinks (soft drinks and artificial juices, such as fruit juice in a box, bottle, or dust).

Each ultra-processed food was evaluated continuously considering the average frequency. To analyze the consumption of ultra-processed foods, a variable was created by combining the evaluated foods. Frequent consumption of ultra-processed foods was defined as eating frequency ≥ 5 days a week [26].

To measure physical inactivity during leisure time, the following questions from Vigitel [24] were used: “In the last three months, did you practice any type of physical exercise or sport?” “What was the main type of physical exercise or sport that you practiced?” “How many days a week do you usually practice physical exercise or sport?” and “On the day you practice exercise or sport, for how long does this activity last?” Physical activity was evaluated using a list of 17 types of physical exercise or sport. For analysis purposes, each physical exercise or sport was categorized according to intensity. Walking, walking on a treadmill, weight training, water aerobics, gymnastics, swimming, martial arts, fighting, cycling, volleyball/footvolley, and dancing were classified as moderate-intensity exercises. In contrast, running, treadmill running, aerobics, soccer/futsal, basketball, and tennis were classified as vigorous-intensity exercises [26].

To determine the physical activity variable, the frequency of practicing each evaluated activity was multiplied by the time in minutes, resulting in the total time spent practicing physical activity during leisure time. Students who practiced moderate-intensity physical activity in their free time for ≥ 150 min/week or vigorous-intensity physical activity for ≥ 75 min/week were classified as “physically active during leisure time.” Contrarily, students who did not practice any physical activity in the last three months or practiced moderate- or vigorous-intensity physical activity less than 150–75 min/week were classified as “physically inactive during leisure time.”

Sedentary behavior was expressed as the total sitting time, defined as follows: “Currently, from Monday to Friday, how many hours on average do you spend sitting (include the time used for cell phone, TV, computer, tablet, books, car, and bus) per day?” This question was adapted from a similar question in the International Physical Activity Questionnaire (IPAQ) [29]. The total sitting time was analyzed as a continuous variable using measures of central tendency. Subsequently, sedentary behavior was established according to the median (p50). Students with daily sitting time < 10 h were considered “non-sedentary,” while those with daily sitting time ≥ 10 h were considered “sedentary.”

### Covariates

Sociodemographic, academic, and health conditions variables were used to describe the sample.

The sociodemographic and academic data used were biological sex (male and female), age (18 to 24 years and > 24 years), skin color (white and non-white—yellow, indigenous, black, brown, or other), sexual orientation (heterosexual and others—homosexual, bisexual, or asexual), marital status (single and others—married, stable union, widowed, or divorced), housing (without family members or with family members), and total family income (1 to 3 minimum wages and ≥ 4 minimum wages). The salary value considered in this study referred to the minimum wage in force in Brazil in 2020 (R$1.045.00). Academic aspects were also evaluated, such as the area of knowledge of the course (life sciences, exact sciences, human, social, and applied sciences) and the period the student was studying (freshmen, veterans, and graduating seniors).

In the health condition domain, the following variables were evaluated: medical diagnosis of anxiety disorders (yes or no) and medical diagnosis of depression disorders (yes or no).

### Statistical analysis

To graphically represent the co-occurrence of obesogenic behaviors, a Venn diagram was prepared using the InteractiVenn program and Microsoft Office Excel.

For sample description and data comparison, the variables were analyzed using frequency distribution and Pearson’s chi-square test, respectively. The proportion and 95% confidence interval (95%CI) were used to estimate the prevalence of anxiety and depression symptoms. Multivariate logistic regression was performed to verify the association between the outcome and explanatory variables. The measure of association used was the Odds Ratio (OR) with their respective 95%CI. Analyses were performed using Stata version 13.0 (Stata Corporation, College Station, TX, USA). Statistical significance was set at 5%.

To select the appropriate adjustment variables, a theoretical model of causality was created using a directed acyclic graph (DAG) considering the outcome (symptoms of anxiety and depression), exposure (co-occurrence of obesogenic behaviors), and possible confounding variables of the explored association. The DAG provides systematic representations of causal relationships, which allows determining sets of covariates to minimize potential confounders through adjustment, using graphical criteria such as the “backdoor” criterion and its extensions [30].

**Fig. 2:**
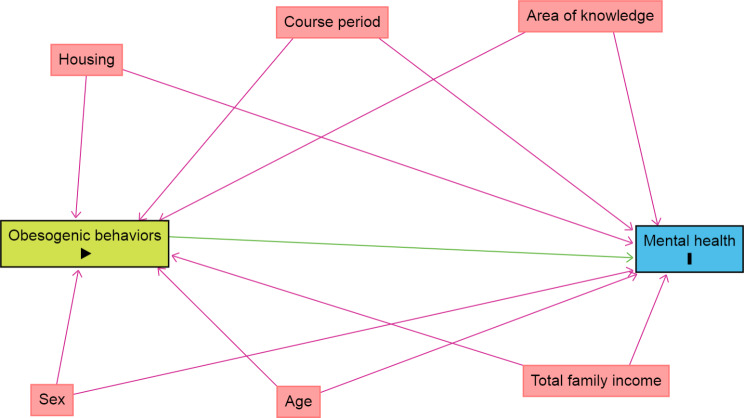
DAG of the association between co-occurrence of obesogenic behaviors and symptoms of anxiety and depression. DAG: Directed acyclic graph. The variable in green and with the “►” symbol inside the rectangle was the explanatory variable; in blue and with the letter “I” inside is the outcome variable, composed of anxiety symptoms and depression symptoms. The fgure shows only the variables that were selected for multivariate. The variables are: sex, age, housing, total family income, course period, and area of knowledge. The arrows indicate the causal relationships between the variables

To elaborate the DAG, the online software Dagitty version 3.0 was used. Causal connections are represented by arrows (Fig. 2). Each DAG variable was chosen based on scientific evidence available in the literature [2, 20, 31].

The variables were represented by rectangles, and the colors had different meanings: blue outlined in black for the outcome variable, green for the exposure variable, blue for the antecedents of the outcome variable, and pink for the antecedents of the outcome and exposure variables.

A minimum set of adjustment variables was defined to avoid unnecessary adjustments, spurious associations, and estimation errors. In addition, the Hosmer–Lemeshow test was used to assess the goodness of fit of the model. Considering the DAG results, the multivariate model was adjusted for sex, age, housing, total family income, course period, and area of knowledge.

## Results

Among the 1,353 students included in this study, the majority were female, aged between 18 and 24 years, non-white, single, heterosexual, living without family members, and with a family income of 1–3 minimum wages. Regarding the course period, most participants was veterans and in the following areas of knowledge: 44.5% in actual sciences, 33.4% in human, social, and applied sciences, and 22.1% in life sciences. 48.3% of the participants reported a medical diagnosis of anxiety, and 23.3% reported a medical diagnosis of depression. The participants’ main characteristics are listed in Table 1.

**Table 1:** Sociodemographic, academic, and health characteristics according to mental health, PADu-COVID Study (2020)

Variables	Total (%)	Anxiety symptoms		*P* value*	Depression symptoms		*P* value*
		Absence^a^ (*n* = 729)	Presence^b^ (*n* = 624)		Absence^a^ (*n* = 729)	Presence^b^ (*n* = 624)	
**Sex**				< 0.001			
Male	456 (33.7)	304 (66.7)	152 (33.3)		243 (53.3)	213 (46.7)	**< 0.001**
Female	897 (66.3)	425 (47.4)	472 (52.6)		371 (41.4)	526 (58.6)	
**Age**				0.214			0.595
18 to 24 years	896 (66.2)	472 (52.7)	424 (47.3)		402 (44.9)	494 (55.1)	
> 24 years	457 (33.8)	257 (56.2)	200 (43.8)		212 (46.4)	245 (53.6)	
**Skin color** ^**c**^				0.244			0.303
White	662 (48.9)	346 (52.3)	316 (47.7)		291 (44.0)	371 (56.0)	
Non-white	691 (51.1)	383 (55.4)	308 (44.6)		323 (46.8)	368 (53.3)	
**Sexual orientation** ^**d**^				**< 0.001**			**< 0.001**
Heterosexual	1.034 (76.4)	608 (58.8)	426 (41.2)		519 (50.2)	515 (49.8)	
Others	319 (23.6)	121 (37.9)	198 (62.1)		95 (29.8)	224 (70.2)	
**Marital status** ^**e**^				0.212			**0.001**
Sigle	1.251 (92.5)	668 (53.4)	583 (46.6)		552 (44.1)	699 (55.9)	
Others	102 (7.5)	61 (59.8)	41 (40.2)		62 (60.8)	40 (39.2)	
**Housing**				0.278			**0.001**
Without family members	853 (63.0)	450 (52.8)	403 (47.2)		359 (42.1)	494 (57.9)	
With family members	500 (37.0)	279 (55.8)	221 (44.2)		255 (51.0)	245 (49.0)	
**Total family income** ^**f**^				**< 0.001**			0.202
1 to 3 minimum wages	733 (54.2)	363 (49.5)	370 (50.5)		321 (43.8)	412 (56.2)	
≥ 4 minimum wages	620 (45.8)	366 (59.0)	254 (41.0)		293 (47.3)	327 (52.7)	
**Course period**				0.649			0.163
Freshmen	178 (13.2)	91 (12.5)	87 (14.0)		72 (11.8)	106 (14.4)	
Veterans	892 (66.1)	482 (66.2)	410 (66.0)		403 (65.7)	489 (66.4)	
Graduating seniors	279 (20.7)	155 (21.3)	124 (20.0)		138 (22.5)	141 (19.2)	
**Area of knowledge**				**< 0.001**			**< 0.001**
Life Sciences	297 (22.1)	184 (62.0)	113 (38.0)		153 (51.5)	144 (48.5)	
Exact Sciences	599 (44.5)	358 (59.8)	241 (40.2)		300 (50.1)	299 (49.9)	
Humanities and Social and Applied Sciences	449 (33.4)	185 (41.2)	264 (58.8)		159 (35.4)	290 (64.6)	
**Medical diagnosis of anxiety disorder**				**< 0.001**			**< 0.001**
No	700 (51.7)	501 (71.6)	199 (28.4)		415 (59.3)	285 (40.7)	
Yes	653 (48.3)	228 (34.9)	425 (65.1)		199 (30.5)	454 (69.5)	
**Medical diagnosis of depression disorder**				**< 0.001**			**< 0.001**
No	1.038 (76.7)	635 (61.2)	403 (38.8)		551 (53.1)	487 (46.9)	
Yes	315 (23.3)	94 (29.8)	221 (70.2)		63 (20.0)	252 (80.0)	

*P value obtained using Pearson’s Chi-Square test; In bold: the statistically significant variables in the bivariate analysis

^a^ Absence: normal and mild symptom categories;

^b^ Presence: moderate, severe, and extremely severe symptom categories;

^c^ Non-white: yellow, indigenous, black, brown, or other;

^d^ Others: homosexual, bisexual, or asexual;

^e^ Others: married, stable union, widowed, or divorced;

^f^ The minimum wage in force in Brazil in 2020 = R$1.045.00

^g^ Freshmen: first semester student; Veterans: students who are enrolled from the second year to the penultimate period of the course; Graduating seniors: last semester student

**Fig. 3:**
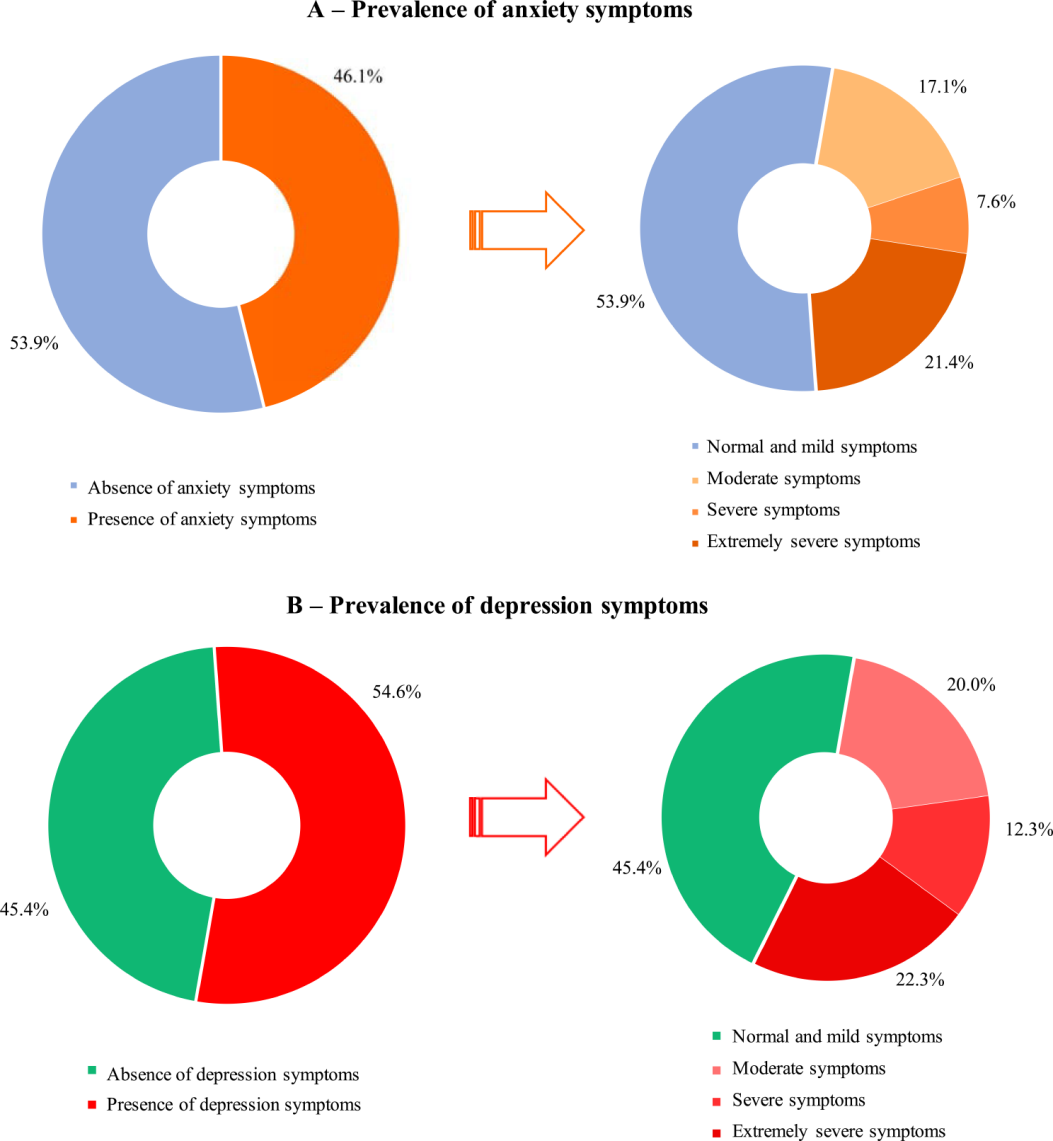
Prevalence of symptoms of anxiety and of depression during the COVID-19 pandemic, PADu-COVID Study (2020)

Figure 3 llustrates the prevalence of anxiety and depression symptoms. Approximately 46.0% of the students had anxiety symptoms and 21.4% had extremely severe symptoms. Depression symptoms were present in 54.6% of the students, and 22.3% were classified as having extremely severe symptoms.

**Fig. 4:**
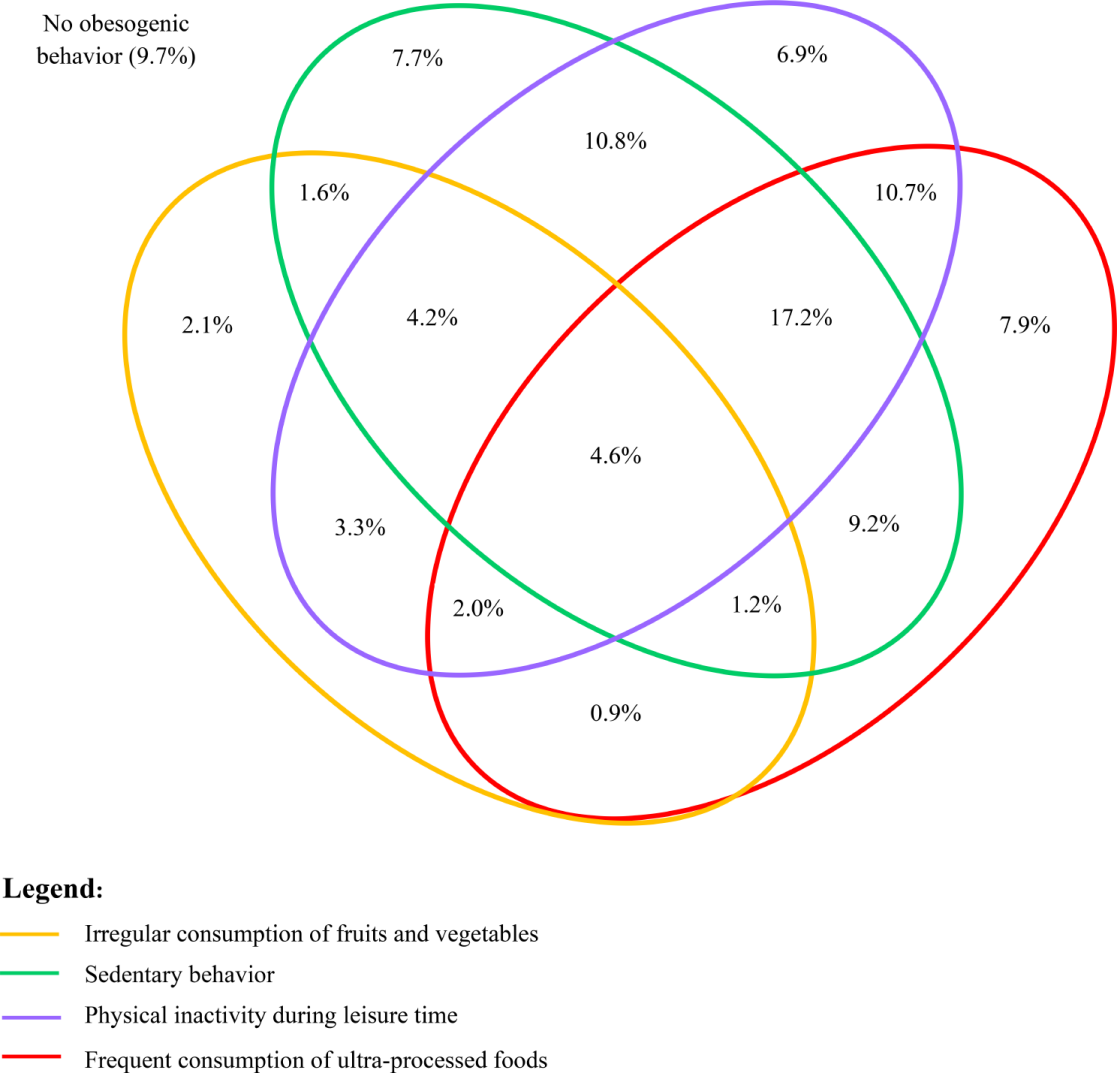
Co-occurrence of obesogenic behaviors during the covid-19 pandemic. PADu-COVID Study (2020) (*N* = 1,209)

Figure 4 shows the co-occurrence of obesogenic behaviors. The Venn diagram shows that the most prevalent combination of obesogenic behaviors is the consumption of ultra-processed foods, physical inactivity during leisure time, and sedentary behavior (17.2%), followed by physical inactivity during leisure time and sedentary behavior (10.8%) and consumption of ultra-processed foods and physical inactivity during leisure time (10.7%). The absence of the four obesogenic behaviors was observed in only 9.7% of the students.

Multivariate regression analysis revealed a dose-response gradient (p-value for linear trend < 0.001); the greater the number of obesogenic behaviors, the higher the chance of anxiety and depression symptoms among the students. The presence of only one obesogenic behavior was not associated with the mental disorders investigated in this study. Students with two obesogenic behaviors were more likely to have anxiety [OR: 1.66 (95% CI: 1.06–2.60)] and depression symptoms [OR: 2.43 (95%CI: 1.57–3.75)] than those who did not show obesogenic behaviors. Students with three or more obesogenic behaviors were more likely to have anxiety [OR: 2.81 (95%CI: 1.77–4.46)] and depression symptoms [OR: 3.46 (95%CI: 2.20–5.43)] than who did not show obesogenic behaviors (Table 2).

**Table 2:** Association between the proportion of obesogenic behaviors and symptoms of anxiety and depression. PADu-COVID Study (2020)

Number of obesogenic behaviors^a^	Prevalence (%)	Presence of anxiety symptoms^b^		*p*-trend*	Presence of symptoms of depression^b^		*p*-trend*
		Unadjusted analysis	Adjusted analysis^c^		Unadjusted analysis	Adjusted analysis^c^	
		OR (95%CI)	OR (95%CI)		OR (95%CI)	OR (95%CI)	
				**< 0.001**			**< 0.001**
**No**	9.7	ref.	-		ref.	-	
**One**	24.6	1.04 (0.66–1.64)	0.92 (0.57–1.48)		1.53 (0.98–2.39)	1.43 (0.91–2.26)	
**Two**	36.5	1.78 (1.16–2.74)	1.66 (1.06–2.60)		2.55 (1.67–3.91)	2.43 (1.57–3.75)	
**Three or more**	29.2	3.15 (2.02–4.90)	2.81 (1.77–4.46)		3.77 (2.42–5.86)	3.46 (2.20–5.43)	

* p-trend referring to the linear trend test;

^a^ The obesogenic behaviors evaluated was: irregular consumption of fruits and vegetables, frequent consumption of ultra-processed foods, physical inactivity during leisure time, and sedentary behavior;

^b^ The categories moderate, severe, and extremely severe indicating the presence of symptoms; the presence of anxiety symptoms and depression symptoms was considered as an outcome;

^c^ Multivariate logistic regression adjusted according to directed acyclic graph. Adjusted for sex, age, housing, family income, course period, and area of knowledge

## Discussion

The results of this study indicated that mental disorders were highly prevalent and multiple obesogenic behaviors were identified among university students during the COVID-19 pandemic. Furthermore, a positive association was observed between the co-occurrence of obesogenic behaviors and symptoms of anxiety and depression, with a dose-response effect.

Entering university is a critical period in terms of psychosocial development, with numerous personal and academic challenges that can increase the risk of mental disorders. The literature points out that psychological suffering does not decrease at any time during graduation, and, in fact, increases as the semesters progress [32, 33]. This fact was observed in the present study, which found a higher prevalence of anxiety and depression symptoms among veteran students when compared to freshmen. Thus, the university environment exposes students to various situations linked to psychological distress, and the COVID-19 pandemic may have exacerbated this scenario, especially for students transitioning from high school to university [34, 35].

Considering the context of the COVID-19 pandemic, epidemiological studies showed a high prevalence of mental disorders among the university population [5, 36, 37]. According to Huckins and collaborators (2020) [38], symptoms of anxiety and depression increased among university students, regardless of their field of study, with the start of the pandemic in 2020 compared to previous periods. A study carried out in Chile at a medium-sized private university using the DASS-21 found that 37.1% of undergraduate students had depressive symptoms, 37.9% symptoms of anxiety and 54.6% symptoms of stress [39].

Evidence showed that the presence of anxiety and depression symptoms during the initial periods of the pandemic was associated with high rates of infection by SARS-CoV-2, decrease in human mobility, which consequently contributed to the lack of interpersonal communication and interaction with colleagues, and suspension of face-to-face classes, which generated changes in the university routine, in addition to fear and uncertainty regarding the future and academic performance [1, 2, 40].

Most universities immediately responded to the public health emergency by suspending face-to-face classes, seeking to minimize the transmission of the virus in the academic environment. However, this sudden change may have directly impacted the mental health and well-being of university students, making them more susceptible to psychological problems such as anxiety, fear, worry, and stress [1, 3, 5, 41].

The preventive measures adopted by countries to reduce exposure and the rate of transmission of the virus led to different lifestyle changes [42, 43]. Evidence suggests that social restrictions during the first months of the pandemic may be associated with obesogenic behaviors, which include changes in eating habits, reduced levels of physical activity, and increased time spent in sedentary behavior [44–48].

In this study, the most prevalent combination of obesogenic behaviors among students was frequent consumption of ultra-processed foods, physical inactivity during leisure time, and sedentary behavior. In the case of university students, remote teaching, as a result of the suspension of face-to-face classes, may have contributed to the growing increase in obesogenic behaviors since they remained seated for long periods, favoring the consumption of quick snacks between meals, and did not commute, both from home to the university and between classrooms [45, 49].

Normally, obesogenic behaviors have a synergistic effect, changing the influence of one behavior on other behaviors, and share contextual determinants, acting directly in developing overweight and obesity or even as mediators of distal determinants that can affect health in the long term [11, 50]. This information has important implications for public health, as it helps identify which obesogenic behaviors cluster together, assisting in developing an integrative approach for effective interventions and initiatives aimed at preventing obesity.

Symptoms of anxiety and depression were associated with the co-occurrence of obesogenic behaviors among students. The greater the number of obesogenic behaviors, the higher the chance of students reporting symptoms of anxiety and depression, which corroborates the findings of Champion et al. [11], Hutchesson et al. [13] and Kwan et al. [15], who observed that students involved in multiple obesogenic behaviors had higher rates of mental health problems, such as anxiety and depression.

One hypothesis that justifies this association is that individuals may engage in more obesogenic behaviors, such as increased consumption of ultra-processed foods and physical inactivity, to help deal with mental health problems [12, 13, 51]. The negative changes related to eating habits can be attributed, for example, to emotional eating or lack of motivation to maintain a healthy diet [46]. In addition, people with a greater perception of stress and depression symptoms tend to increase the consumption of sugary foods to deal with these symptoms [51, 52].

There is evidence that shows that mental disorders and obesity have a bidirectional relationship, that is, these conditions can predict and can be predicted by the occurrence of the other. In other words, obesity can predict and be predicted by the occurrence of mental disorders, and vice versa [52–54]. The association between mental health and obesity can be explained, at least in part, by the direct effect of cortisol, one of the main hormones involved in the biological response to stress, the storage of abdominal fat and the increased intake of unhealthy foods [52, 55]. Mental health problems can also interfere with the adoption of healthy lifestyle behaviors, while promoting obesogenic behaviors, such as physical inactivity, which are related to the development of obesity [52].

Although the findings of this study are consistent with those reported in the literature, some limitations must be considered. First, studies that assess the presence of mental disorders among university students use different measurement instruments and cutoff points, which limit data comparison. In this study, a self-reported scale was used to assess the presence of symptoms and was not based on a medical diagnosis. In addition, the symptom classifications of mental disorders investigated in this study may differ from those of other national and international studies. Another limitation is the inability to infer causality between obesogenic behaviors and symptoms of anxiety and depression due to the cross-sectional design of the study. It is important to emphasize that this study was based on self-reported obesogenic behaviors, which may have led to information bias, since young people tend to overestimate or, at other times, underestimate exposure to obesogenic behaviors. The findings of this study are consistent with those in the literature.

Despite the limitations mentioned, the results obtained add important evidence on the prevalence of mental disorders and obesogenic behaviors, as it expands scientific knowledge, mainly by considering behaviors that are deleterious to health evaluated simultaneously. To the best of our knowledge, this is the first study to evaluate the association between the presence of anxiety and depression symptoms and the simultaneity of different obesogenic behaviors among university students during the COVID-19 pandemic in Brazil. The epidemiological panorama of studies like this, as well as the analysis of the simultaneity of different obesogenic behaviors deserve to be highlighted, since much of the research tends to analyze them in isolation, as if they were completely independent. However, it is necessary to consider that these behaviors are complex and interrelated, increasing the risk of developing chronic diseases.

## Conclusion

The results of this study suggest that symptoms of anxiety and depression are highly prevalent among university students and that the greater the number of obesogenic behaviors, the higher the chance of symptoms of mental disorders.

The positive relationship observed between mental health disorders and the co-occurrence of obesogenic behaviors highlights the importance of promoting physical and mental health in the university environment. The results of the present study suggest that promoting a healthy lifestyle would help reduce obesogenic behaviors, decreasing the risk of developing several chronic diseases and mental disorders. Therefore, the findings have important implications for public health, as they can help in the development of comprehensive and integrative public initiatives and policies aimed at preventing obesity.

By understanding how anxiety and depression symptoms are associated with different obesogenic behaviors, these findings highlight the potential for universities to adopt mental health promotion strategies and actions aimed at specific groups of students who may be at higher risk of mental health problems. In addition, since the university represents an opportune environment for health promotion, institutions should focus on creating an environment that motivates healthy habits and promotes, encourages, and supports health-protective behaviors, such as physical activity and healthy eating.

## Data Availability

The datasets generated and/or analyzed as part of the current study are not publicly available due to confidentiality agreements with subjects. However, they can be made available solely for the purpose of review and not for the purpose of publication from the corresponding author upon reasonable request.
